# The Role of Clinical Proteomics, Lipidomics, and Genomics in the Diagnosis of Alzheimer’s Disease

**DOI:** 10.3390/proteomes4020014

**Published:** 2016-03-31

**Authors:** Ian James Martins

**Affiliations:** School of Medical Sciences, Edith Cowan University, 270 Joondalup Drive, Joondalup 6027, Australia; i.martins@ecu.edu.au; Tel.: +61-8-6304-2574

**Keywords:** diagnosis, biomarkers, Alzheimer’s disease, lipidomics, genomics, chronic disease, plasma, cerebrospinal fluid

## Abstract

The early diagnosis of Alzheimer’s disease (AD) has become important to the reversal and treatment of neurodegeneration, which may be relevant to premature brain aging that is associated with chronic disease progression. Clinical proteomics allows the detection of various proteins in fluids such as the urine, plasma, and cerebrospinal fluid for the diagnosis of AD. Interest in lipidomics has accelerated with plasma testing for various lipid biomarkers that may with clinical proteomics provide a more reproducible diagnosis for early brain aging that is connected to other chronic diseases. The combination of proteomics with lipidomics may decrease the biological variability between studies and provide reproducible results that detect a community’s susceptibility to AD. The diagnosis of chronic disease associated with AD that now involves genomics may provide increased sensitivity to avoid inadvertent errors related to plasma *versus* cerebrospinal fluid testing by proteomics and lipidomics that identify new disease biomarkers in body fluids, cells, and tissues. The diagnosis of AD by various plasma biomarkers with clinical proteomics may now require the involvement of lipidomics and genomics to provide interpretation of proteomic results from various laboratories around the world.

## 1. Introduction

In the world approximately 44 million people have been diagnosed with Alzheimer’s disease (AD) or related dementia. The global cost of AD and dementia has now been estimated to be approx. $605 billion and is equivalent to 1% of the world’s gross domestic product. In the United States AD rates by age classifications are: 85+ years, 38%, 75–84 years, 44%, 65–74 years, 15%, <65 years, 4%. The age group under 65 years has been diagnosed with approx. 4% of the AD cases and as of 2016 the susceptibility to AD may have originated early in life with chronic diseases such as obesity, diabetes, and neurodegenerative diseases closely associated with hypothalamic disturbances and neurodegenerative diseases. AD is a neurodegenerative condition that involves disturbances in multiple higher brain functions including memory and cognition. Amyloid beta is the main constituent of AD senile plaques [1] and prevention of amyloidosis and dementia may require the diagnosis of individuals early in life to link chronic disease progression with early neurodegeneration.

**Figure 1:**
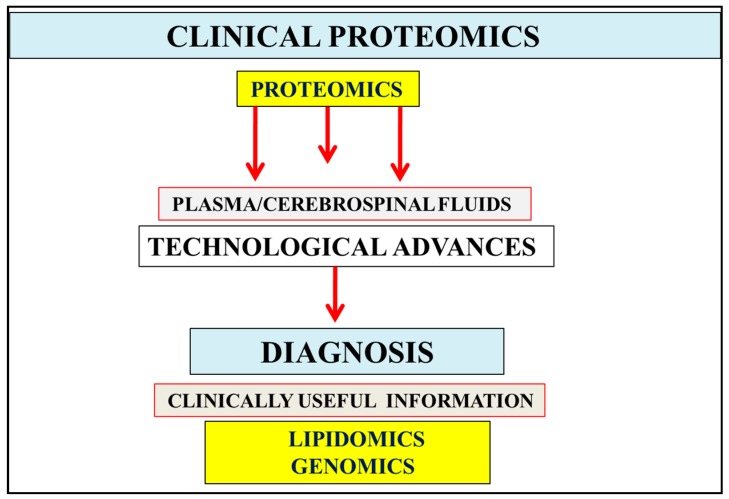
In body fluids such as the urine, plasma, and cerebrospinal fluids, the abundance of proteins has become of importance in the diagnosis of various chronic diseases including Alzheimer’s disease. Testing of these fluids involves biological variation or differences in the sample along with inter- and intra-assay variability that may influence proteomics results. Measurement and data analysis of fluid proteomics can be improved by comparison with genomic and lipidomic data. Technological advances in Alzheimer’s disease (AD) allow interpretation of clinical proteomics, lipidomic, and genomic data that may assist in the development of new protein biomarkers with relevance to the reversal of chronic disease and the diagnosis of AD.

In the current global epidemic the incidence of obesity and diabetes has been associated with non-alcoholic fatty liver disease (NAFLD) and insulin resistance, which in the developing and developed world may rise to between 30% and 40% of the global population [1,2]. Individuals homozygous for apo E4 develop AD but non-apo E4 individuals may also develop AD later in life, linked to chronic disease progression early in life involved in the induction of these AD cases (75–84 years, 44% AD cases). The association between chronic disease progression and AD indicates that clinical proteomics (Figure 1) may provide novel biomarkers with a serum protein profile that may diagnose early progression to later-life AD. Plasma biomarkers such as amyloid beta have been an important diagnostic biomarker for AD but non-reproducible and insensitive results for amyloid beta have been obtained for the diagnosis of AD [3,4,5].

The cholesterol–AD connection [6] has attracted considerable interest with relevance to treatment with nutritional diets that maintain cell cholesterol homeostasis. The calorie-sensitive anti-aging gene Sirtuin 1 (Sirt 1) is closely involved in global disease progression with accelerated NAFLD, adiposity, and AD closely linked to Sirt 1 downregulation, hypercholesterolemia, and dementia [1,2]. Sirt 1 is one of the nuclear receptors that is known to regulate several cell functions by deacetylating both histone and non-histone targets. Sirt 1 is an NAD (+)-dependent class III histone deacetylase protein that targets transcription factors to adapt gene expression to metabolic activity, insulin resistance, and inflammation in chronic diseases. Dyslipidemia in AD has now become relevant to amyloidosis with tissue lipidomic analysis that indicates elevation in sphingolipids (sphingosine 1 phosphate) and ceramides in insulin-resistant and AD individuals [1]. Membrane cholesterol and sphingomyelin interactions are critical to amyloid beta oligomer formation with increased cellular ceramide levels associated with cholesterol displacement in membranes with relevance to amyloidosis and AD [7,8,9]. The interest in clinical lipidomics in AD has accelerated in recent years with relevance to early diagnosis by lipidomics of AD when compared with protein biomarker studies and may provide reproducible results for late MCI, prodromal disease, and dementia [10,11,12]. Analysis of a spectrum of plasma, tissue, or cerebrospinal fluid lipids (fatty acyls, glycerolipids, glycerophospholipids, sphingolipids, sterols, and prenols) assists in the diagnosis of various neurological diseases such as AD that are linked to chronic diseases in global populations [13,14,15,16,17,18]. Furthermore, new blood biomarker lipidomic panels have been identified with diagnostic value relevant to the preclinical and late stages of AD [19,20].

The global deterioration scale created by Reisberg provides the stages of cognitive function for those suffering from a primary degenerative dementia such as AD [11]. Stages 1–3 are the pre-dementia stages and stages 4–7 are the dementia stages. Lipidomic and genomic information have become important to the diagnosis of the early stages [1,2,3] of AD (Figure 1), with abnormalities in cholesterol and lipoprotein metabolism [1] closely linked to the late progression of AD with changes in cognition or behavioral symptoms. Nutritional interventions for AD treatment and prevention in stages 1–3 have become important as the neuron disease that involves nuclear membrane changes induced by unhealthy diets and toxins is related to the corruption of the plasma and cerebrospinal fluid (CSF) dynamics, which may be reversible in early stages of the disease [21]. Plasma biomarker measurements by lipidomics and genomics (Figure 1) may provide more sensitive information compared with CSF measurements that may lead to erroneous diagnostic interpretations with lack of external quality assessment [22].

Information obtained from lipidomic and genomic analysis may provide information in relation to hepatic cholesterol metabolism, which is abnormal early in chronic disease and associated with neurodegeneration and biomarkers now relevant to diagnosis of early brain changes in individuals susceptible to AD. The use of lipidomic technology allows the study of the lipid composition of tissues such as the liver and brain that may determine the peripheral metabolism of cholesterol and amyloid beta that are important to the early stages of AD [23]. The brain–liver crosstalk is now central to the metabolism of amyloid beta, with the involvement of adipose tissue of relevance to hepatic amyloid beta metabolism [2]. Therefore, lipidomic and genomic tests for diagnosis of AD indicate that nuclear receptors are relevant to connections between insulin resistance, chronic disease, and AD. Downregulation of liver and brain cell nuclear receptors such as Sirt 1 in stages 1–3 of AD [11] is linked to chronic diseases (obesity/diabetes) and responsible for glucose, cholesterol, and amyloid beta metabolism disorders, which are abnormalities in the early stages of AD.

## 2. Multifactorial Nature of Alzheimer’s Disease Provides Important Links to Early Diagnosis

**Figure 2:**
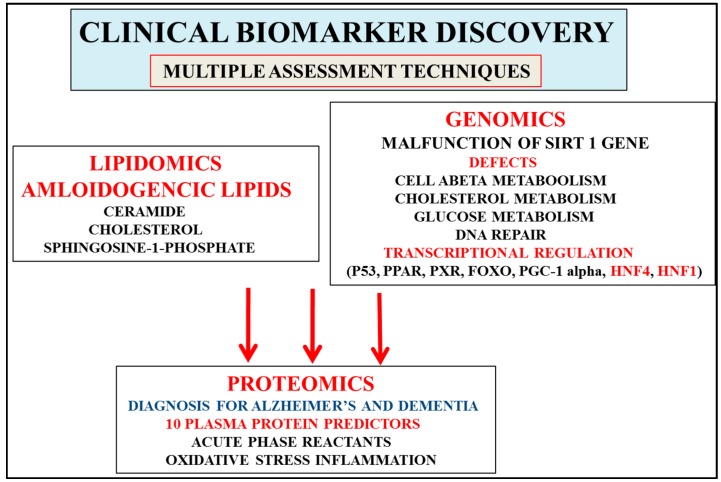
Discovery of biomarkers that are consistent with the stages of development of AD and involve progression that includes mild cognitive impairment, prodromal disease, and the development of dementia. Proteomics that involve 10 plasma protein predictors (AD/dementia) and acute phase proteins (MCI/prodromal disease) allow detection of the severity of the stages of AD. Interest in genomics has accelerated with the identification of the calorie-sensitive gene Sirt 1, which may allow early AD diagnosis when compared to relevant proteomic/lipidomic biomarkers (ceramides, sphingolipids) that are involved in the later stages of AD. Interventions with nutritional therapy may activate cell Sirt 1/transcriptional regulation and maintain glucose, cholesterol, and amyloid beta levels connected to the delay in the progression and severity of AD (stages 4–7). Furthermore, multiple technologies may separate and diagnose individuals with other neurological disease from AD that may involve plasma, CSF, and tissue analysis.

Proteomics has become important to the understanding of disease diagnosis with the apo E isoforms (apo E2, E3, and E4) related to the increased risk for AD [21]. Apo E4-related diseases have been shown to initiate toxic events that lead to synaptic dysfunction and neurodegeneration in AD [24]. The multifactorial nature of AD has raised concerns since apo E4 has also been shown to be involved in various other diseases such as insulin resistance, cardiovascular disease, hypercholesterolemia, obesity, and NAFLD [25,26,27,28]. Apo-E isoforms regulate Aβ aggregation and clearance in the liver and brain with effects of brain lipid transport, glucose metabolism, neuroinflammation, and mitochondrial function on amyloid beta transport [24]. The role of apo E4 in chronic disease progression is well understood but its role may be secondary compared to the primary role the anti-aging protein Sirt 1 has in nuclear receptor and transcriptional regulation involved with NAFLD, insulin resistance, mitochondrial function, neurodegeneration, cardiovascular disease, and AD [2,29,30,31,32,33,34,35]. Individuals with apo E3 have become of major concern for insulin resistance and NAFLD with links to AD [1,2] since NAFLD has risen to 30% of the global population and a lack of hepatic Sirt 1 activity relevant to the defect in the peripheral sink beta clearance pathway [23,36,37] associated with accelerated brain amyloidosis [36]. Diagnosis of early AD and neurological disease now involves clinical genomic cell analysis with Sirt 1 (Figure 2), transcriptional dysregulation [2,36,37] involved in appetite, and metabolic disease and obesity with relevance to hepatic amyloid beta, cholesterol, and glucose metabolism.

Clinical genomic testing (apo E) plays a major role in the diagnosis of AD and avoids inadvertent errors related to plasma *versus* CSF testing by proteomics and lipidomics that identify early and novel disease biomarkers in body fluids, cells, and tissues. Nutritional regulation of Sirt 1 in peripheral cells determines the peripheral clearance pathways for amyloid beta involving apolipoprotein E (apo E) and albumin [38,39]. Brain amyloid beta clearance [21,36] is secondary to nutritional regulation with the role of the liver Sirt 1 central and important to spontaneous brain abeta aggregation (oligomers and fibrils). Sirt 1 gene expression (Figure 2), especially its effects on transcriptional regulation and DNA methylation [2,35,40] in cells, has become of importance and may supersede apo E genotyping in cells for differential diagnosis of early AD.

**Figure 3:**
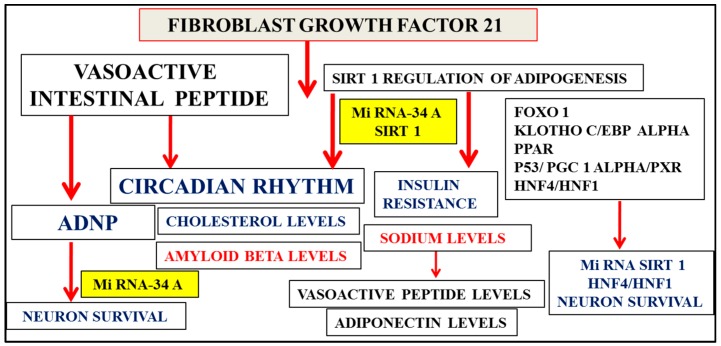
Fibroblast growth factor 21 (FGF21) regulates Sirt 1-mediated release of adiponectin from adipose tissue with relevance to NAFLD and the metabolic syndrome. Micro RNA, Sirt 1, and transcription factor interactions are possibly involved in vasoactive intestinal peptide (VIP)-mediated release of ADNP from neurons with critical links of Sirt 1 and ADNP in neuron survival. FGF21, Sirt 1, and VIP are associated with the circadian brain–liver amyloid beta clearance pathway with decreased VIP levels in plasma associated with excess sodium intake and sodium intake linked to adiponectin levels. Sirt 1’s downregulation, associated with insulin resistance, is linked to high plasma sodium levels and low plasma VIP levels. This is related to its vascular effects in the central nervous system, which determine hepatic amyloid beta metabolism and the regulation of apo E-mediated amyloid beta clearance pathways in the brain.

Sirt 1 increases adiponectin transcription in adipocytes [2] by activation of forkhead transcription factor O1 (Foxo) interaction with CCAAT/enhancer-binding protein alpha (C/EBPalpha) to form a transcription complex at the mouse adiponectin promoter that upregulates adiponectin gene transcription [41]. Sirt 1 interactions with C/EBPalpha may involve Klotho C/EBPalpha and peroxisome proliferator-activated receptor (PPAR) interactions [2,42,43,44] with their important role in adipocyte differentiation. Dietary downregulation of Sirt 1 contributes to reduced adiponectin expression in obesity and diabetes [41] with effects on adipose tissue transformation and liver development [45]. Fibroblast growth factor 21 (FGF21) is an important activator of Sirt 1-mediated release of adiponectin [46]. FGF21 binds to FGF receptor and beta koltho receptor complex [47,48,49,50,51] and activates adipose tissue Sirt 1 by increases in NAD+ and activation of peroxisome proliferator-activated receptor gamma coactivator 1-alpha (PGC1-alpha) and AMP-activated protein kinase (AMPK) [46,52]. Nutrition and PPAR alpha-Sirt 1 expression related to hepatic FGF21 production has become important to NAFLD and the metabolic syndrome [53,54,55,56,57]. FGF21 is regulated by fasting and feeding and with vasoactive intestinal peptide (VIP) associated with the circadian brain–liver amyloid beta clearance pathway [51,58,59,60,61] (Figure 3).

Low adiponectin levels and hypercholesterolemia with low high density lipoproteins (HDL) apolipoprotein AI levels and high low density lipoprotein (LDL) apolipoprotein B levels have been associated with insulin resistance and AD (Figure 3) [1]. Research into adiponectin and its regulation of ceramide metabolism (Figure 2) has shown that the effects on sphingolipid (sphingosine-1-phosphate) metabolism are connected to pancreatic insulin production [62,63,64,65]. Adiponectin deficiency has been shown to reduce hepatic ATP-binding cassette transporter ABCA1 (ABCA1) and apo AI synthesis with relevance to the reverse cholesterol transport [66]. Hepatic FGF21 has been shown to regulate lipolysis (fatty acid release) with FGF21 critical in the reduction of adipose tissue ceramides. In insulin resistance and AD, FGF21 and adiponectin levels are implicated in increased cellular ceramide levels associated with cholesterol displacement in membranes with relevance to amyloidosis and AD [7,8,9]. Sirt 1/adiponectin/FGF21 dysregulation determine hepatic cholesterol metabolism with effects on plasma apo B levels mediated via Sirt 1 and transcription factor C/EBPalpha, which regulates the transcription of the apo B gene [67]. Sirt-regulated transcription factors such as hepatocyte nuclear factor 4 (HNF4) and PGC1 alpha have been shown to modulate hepatic apolipoprotein synthesis with relevance to hepatic lipid metabolism [68,69,70,71].

Sirt 1 gene expression and its regulation early in life is central to hepatic glucose, cholesterol, and amyloid beta metabolism and their downregulation in stages 1–3 of AD (Figure 2) avoid errors with relevance to proteomic (adiponectin, FGF21) and lipidomic (ceramides, sphingolipids) analysis that may show increased or altered levels in plasma and CSF in later stages 4–7 of AD. Sirt 1’s role in circadian rhythms [72,73] and neuron survival is shown by connections to the circadian neuron regulation by the neuropeptide vasoactive intestinal peptide (VIP) [74,75,76,77] mediated via the activity-dependent neuroprotective protein (ADNP) release from astrocytes and the protection of neurons (Figure 3) [78,79,80,81,82,83]. Decreased VIP levels in plasma are associated with excess sodium intake [84,85] and sodium intake linked to adiponectin levels [86]. Sirt 1’s links to insulin resistance (Figure 3) are associated with high plasma sodium levels [87,88,89] and low VIP levels, which contribute to coronary events [90] and vascular effects in the central nervous system that determine peripheral amyloid beta metabolism and transport of amyloid beta across the blood–brain barrier [91,92] and are important to the role of VIP in the regulation of apo E-mediated amyloid beta clearance pathways in the brain and the liver [21,23,93].

Micro RNAs and neuron survival have become important, with micro RNA 34a (miR 34a), which inhibits Sirt 1, relevant to metabolic diseases and neurogenesis (Figure 3), possibly through interactions with hepatocyte nuclear factor (HNF4/HNF1) alpha (MODY gene), which may be relevant to the diagnosis of NAFLD and neurodegenerative diseases [2,94,95,96]. Interference of HNF4/PGC1 alpha by the transcription factor pregnane X receptor (PXR) is possibly connected to Sirt 1 regulation of PXR-mediated modulation of HNF4/PGC1 alpha, which is important for drug and cholesterol metabolism [18,97]. Sirt 1 deacetylation of the transcription factor p53 [2] mediates the effects of PXR with respect to HNF4/PGC1 alpha regulation [98,99] of hepatic cholesterol metabolism and amyloid beta metabolism, and also involves p53’s effects on VIP with ADNP release from neurons (Figure 3) [100]. Nutritional regulation and insulin resistance, which rely on miR 34a/Sirt 1 involvement in HNF-1/HNF4 interactions [101,102], are central to the links between the genetic regulation of diabetes and neurogenesis [36,96,103].

Interest in the field of proteomics has accelerated to determine the plasma biomarkers that provide increased sensitivity so as to avoid inadvertent errors related to plasma *versus* CSF markers for the reproducible diagnosis for AD. Biomarker studies that are consistent with the stages of development of specific brain changes in AD involve progression from no clinical manifestation to a prodromal stage with mild cognitive impairment and the development of final prodromal disease with dementia [10,11,12]. The plasma biomarkers involved in oxidative stress and inflammation that may be important to early diagnosis for AD (no clinical manifestation) are the hepatic acute phase reactants/cytokines (APP) involved in amyloid beta homeostasis with corruption of apo E-mediated cholesterol transport [21,23]. Acute phase proteins that directly interact with amyloid beta oligomers include serum amyloid protein P, serum amyloid protein A, alpha 2 macroglobulin, gelsolin, complement components, transthyretin, and clusterin [21]; these biomarkers assist with the early diagnosis of AD (Figure 2).

In a multiplexing approach a plasma protein panel has been identified to assess disease severity for predicting disease progression from prodromal disease to dementia. Ten proteins have been identified to diagnose AD [10]: include transthyretin, clusterin, cystatin C, alpha 1 acid glycoprotein, intercellular adhesion molecule 1, complement C4 , pigment epithelium-derived factor, alpha 1 antitrypsin, RANTES, and apolipoprotein C3. These plasma protein biomarkers important to AD diagnosis overlap with CSF protein measurements in other neurological diseases [104,105,106,107,108,109,110,111,112]. Other novel proteomic candidate markers have been identified recently and promise to diagnose the very early and late stages of AD [113]. Furthermore, the activity-dependent neuroprotector homeobox protein has been shown to be downregulated in AD and may also be an important diagnostic marker for AD [81]. Proteomics that involve clinical biomarker discovery allow detection of severity for AD but the specific role of genomics such as cellular Sirt 1 may be to allow early diagnosis with Sirt 1 activation associated with relevant proteomic biomarkers (Figure 2) involved in the delay of the severity of disease progression from MCI to prodromal disease and dementia [114,115,116,117,118,119].

Nutritional research, involved in the activation of hepatic Sirt 1, which increases the low adiponectin levels associated with metabolic syndrome, platelet aggregation, and angiogenesis [119,120,121,122,123], is now required. Adiponectin has been shown to form protein complexes with alpha2-macroglobulin and thrombospondin-1 (TSP-1). Alterations in adiponectin levels determine alpha2–macroglobulin–amyloid beta interactions and TSP-1–amyloid beta interactions [124,125,126,127], which involve binding to cell receptors such as low-density lipoprotein receptor-related protein and heparin sulphate proteoglycans [128,129], showing a relationship between TSP-1 and amyloid beta in post-prandial lipid metabolism [37]. Adiponectin is important in astrocyte-neuron amyloid beta metabolism [1], with the effects of adiponectin on brain–liver amyloid clearance determined by proteins such as alpha 2 macroglobulin and TSP-1. TSP-1 is a matricellular protein involved in inflammation and in interactions with various proteins, platelet aggregation, and nitric oxide dyshomeostasis associated with cardiovascular disease, stroke, and diabetes [129,130,131,132,133,134,135,136,137,138,139]. TSP-1 is important to neuron synaptogenesis, with astrocyte TSP-1 release [125] determined by brain adiponectin content with effects on astrocyte-neuron amyloid beta clearance [1]. Genomics and proteomics may assist in the early diagnosis of AD through the primary role of the gene Sirt 1, adiponectin, and TSP-1 in amyloid beta homeostasis, while the corruption of liver and brain regulation has an effect on amyloid beta metabolism [21,23].

TSP-1 regulates transforming growth factor beta (TGF-β) levels with effects on TGF-β signaling, which determines TGF-β partitioning between lipid raft/caveolae- and clathrin-mediated endocytosis pathways [140,141,142,143,144]. Interactions between TSP-1 and TGF-β determine cell liver cholesterol (post-prandial lipid metabolism) [37] and peripheral amyloid beta homeostasis, which are relevant to chronic liver disease, NAFLD, atherogenesis, brain apoptosis, and AD [145,146,147,148,149,150,151]. The anti-aging protein GDF11, which belongs to the TGF-β family, has been shown to restore muscle and brain function [152,153], while the role of TSP-1 in the regulation of GDF11 needs to be determined.

## 3. Nutriproteomic Diets Regulates Plasma Biomarkers and Reverses Neurodegeneration and Amyloidosis

Clinical biomarker discoveries have become important with nutriproteomics [154,155,156] as a technology that could determine biomarkers that may assist with the maintenance of normal cognitive development in individuals at risk of AD. The ingestion of nutrients allows proteomic tools to characterize molecular and cellular changes in protein expression and function in the plasma and CSF [157,158,159,160] with respect to nutritional diets that activate the anti-aging gene Sirt 1 and allow proteins such as amyloid beta/alpha synuclein to maintain monomer interactions and prevent self-association that induces inflammation [38,39]. Low-calorie diets (glucose, fatty acids) regulate Sirt 1/adiponectin expression and nuclear interactions that involve nuclear receptors, transcription factors, and microRNAs that determine liver apo AI/apo B kinetics and ceramide metabolism with respect to amyloid beta metabolism in non-diabetic and diabetic individuals [161]. Integration of proteomics, lipidomics, and genomics technologies allows for the interpretation of various nutritional and dietary interventions that assist in the reversal of neurodegeneration in late MCI, prodromal disease, and dementia.

**Figure 4:**
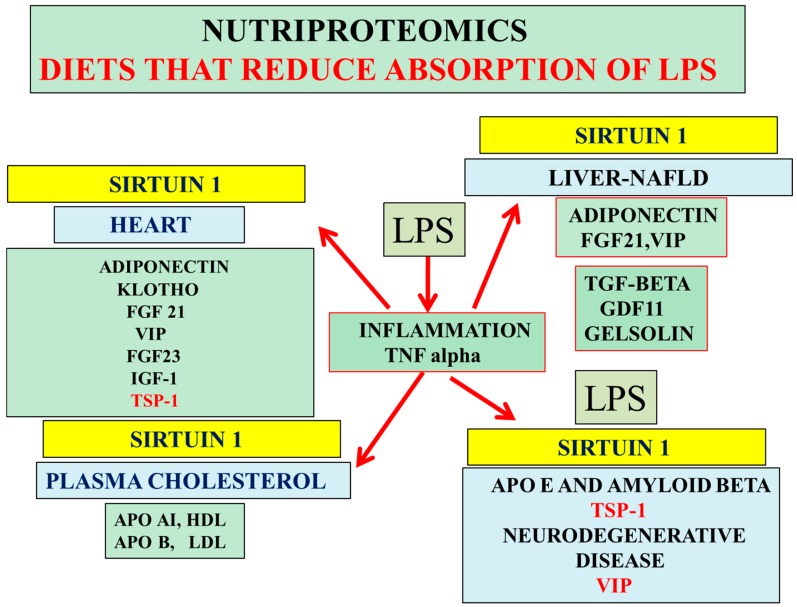
Nutriproteomic diets as a treatment for cardiovascular disease, NAFLD, and AD activate hepatic Sirt 1 and increase hepatic FGF21 and VIP levels relevant to the maintenance of the hepatic peripheral amyloid beta clearance pathways. LPS suppresses Sirt 1/adiponectin expression and increases TSP-1 release from cells with effects on adiponectin and peripheral amyloid beta/cholesterol clearance pathways. Nutritional therapy reduces LPS-induced inflammation and maintains hepatic TGF-β/cholesterol levels (apo AI, apo B); these nutriproteomic diets avoid the transfer of LPS/mycotoxin from the blood plasma to the CSF to prevent LPS/mycotoxin-induced brain apo E neutralization associated with the prevention of amyloidosis and neurodegeneration. Low calorie diets maintain plasma anti-aging protein GDF11, FGF21, VIP, gelsolin, insulin like growth factor 1 (IGF-1), and klotho levels, which are relevant to chronic diseases and AD.

Nutritional research (low fat diets) that targets the intestine lowers the absorption of bacterial lipopolysaccharides (LPS) that have effects on acute inflammation, which involves lymphocytes, monocytes, and macrophages that stimulate tumor necrosis factor alpha (TNF-α) secretion from cells [162]. LPS induces NAFLD and insulin resistance, with insulin resistance linked to alterations in plasma/CSF sodium levels [163] (Figure 4). LPS alter apo E and amyloid beta interactions with accelerated amyloidosis [37,39,162,164] associated with biomarkers such as hepatic cytokines and APP-associated inhibition of reverse cholesterol transport [1,23]. Nutriproteomic diets, such as high fiber diets [6,135], have become important now that inflammatory regulation associated with LPS repression of Sirt 1 [21,37] has been linked to atherogenic diets. Nutriproteomic diets such as very low carbohydrate diets maintain the circadian rhythm, brain–liver amyloid beta pathways, and sodium balance [165], reverse cholesterol transport involving adiponectin [86,166,167], and prevent cognitive decline, cardiovascular disease, and diabetes. These diets involve the measurements of plasma/CSF levels of adiponectin, FGF21, VIP, Klotho, IGF-1, TSP-1, TGF-beta, and gelsolin and may be relevant to healthy dietary interventions (Figure 4).

Interventions with nutriproteomic diets in individuals with MCI to prodromal disease and dementia may allow activation of Sirt 1/adiponectin expression with the identification of changes in plasma/CSF biomarkers (Figure 3) relevant to treatment of individuals with severe forms of prodromal disease and dementia. Furthermore, high fat diets increase adipose tissue TSP-1 levels [168,169] and thus the risk for cardiovascular disease, neuron dysfunction, and defective NO pathways. Sirt 1 regulation of endothelial NOS has been reported with respect to neuroprotection and vascular-related diseases [170]. Diets that reduce TSP-1 improve adipose tissue apelin/Sirt 1 effects on nitric oxide disturbances and vasoconstriction [170]. Angiotensin II is critical to the regulation of TSP-1 levels in cells [171,172], with apelin/angiotensin II/TSP-1 interactions affecting toxic amyloid beta generation [170]. The effects of TSP-1 on inflammation involve TNF-α [173,174,175], which is relevant to the treatment of cognitive impairment and neuron dysfunction [176,177]. Adiponectin expression and TNF-α expression are connected with low adiponectin levels, which are associated with high TNF-α levels [178]. Adiponectin pretreatment has been shown to reduce hepatic TNF-α levels with inhibition of LPS-induced effects in the liver [179] and maintenance of apo E activity [162,164] involved in the reduction of TNF-α secretion [180,181,182]. Low fat diets are possibly important in the maintenance of hepatic Sirt1/adiponectin expression, with inhibition of LPS/TNF-α effects and maintenance of FGF21 effects in the adipose tissue [183,184,185,186] and VIP effects in the liver (apo E mediated) allowing for the clearance of peripheral amyloid beta [51].

Diets that contain zinc may maintain Sirt 1 expression [21] and prevent abnormal LPS/zinc interactions. Zinc is sensitive to HNF4 [187] and reduced hepatic TNF-α toxicity [188,189] may aid the prevention of insulin resistance and AD [1]. Nutriproteomic diets as a treatment for reduction of LPS effects may lower TSP-1 expression by peripheral cells [190,191,192] and improve peripheral LPS–protein interactions [193,194,195,196] to accelerate peripheral amyloid beta metabolism with respect to treatment of individuals with prodromal disease and dementia. Nutritional diets that activate Sirt 1 and maintain therapeutic VIP and FGF21 levels [51] accelerate hepatic LPS/mycotoxin metabolism without transfer to the CSF and brain compartment [21], which is consistent with the use of nutritional therapy to maintain CSF composition, recycling, and amyloid beta dynamics [197,198] for the prevention of early neurodegeneration (stages 1–3) and amyloidosis.

## 4. Conclusions

The susceptibility to AD earlier in life may now involve chronic diseases such as obesity, diabetes, and neurodegenerative diseases. Nutritional interventions and early diagnosis may reduce spontaneous amyloid beta oligomerization associated with the excessive global cost ($605 billion) of late onset AD (>65 years). In the global crisis the inflammatory effects of LPS on apo E/Sirt 1 neutralization reaction induce increased TSP-1 levels that may be relevant to multifactorial diseases including cardiovascular diseases, NAFLD, and neurodegenerative diseases. Nutritional interventions are required to reduce the absorption of LPS early in life to prevent induction of inflammation linked to circadian abnormalities, accelerated amyloidosis metabolic disease, and neurodegeneration. Technologies of lipidomics, genomics, and proteomics are required to assess early plasma lipid and protein biomarkers that indicate repression of nuclear Sirt 1, which involves oxidative stress, inflammation associated with programmed cell death in chronic diseases, and early stages of AD (Reisberg). The genomic, lipidomic, and proteomic interpretation may provide evidence that apo E/Sirt 1 repression and liver disease in global populations is the major defect in early and late stages of MCI with links to prodromal disease and dementia.

## References

[1] Martins I.J., Creegan R. (2014). Links between Insulin Resistance, Lipoprotein Metabolism and Amyloidosis in Alzheimer’s Disease. Health.

[2] Martins I.J. (2015). Unhealthy Nutrigenomic Diets Accelerate NAFLD and Adiposity in Global communities. J. Mol. Genet. Med..

[3] Tamaoka A.T., Fukushima N., Sawamura K., Ishikawa E., Oguni Y., Komatsuzaki Y., Shoi S. (1996). Amyloid beta protein in plasma from patients with sporadic Alzheimer’s disease. J. Neurol. Sci..

[4] Vanderstichele H.E., van Kerschaver C., Hesse P., Davidsson M.A., Buyse N., Andreasen N., Minthon L., Wallin A., Blennow K., Vanmechelen E. (2000). Standardization of measurement of β-amyloid_(1–42)_ in cerebrospinal fluid and plasma. Amyloid.

[5] Mayeux R., Tang M.X., Jacobs D.M., Manly J., Bell K., Merchant C., Small S.A., Stern Y., Wisniewski H.M., Mehta P.D. (1999). Plasma amyloid β-peptide 1–42 and incipient Alzheimer’s disease. Ann. Neurol..

[6] Martins I.J., Fernando W. (2014). High Fibre Diets and Alzheimer’s Disease. Food Nutr. Sci..

[7] Yu C., Alterman M., Dobrowsky R.T. (2005). Ceramide displaces cholesterol from lipid rafts and decreases the association of the cholesterol binding protein caveolin-1. J. Lipid Res..

[8] Ali M.R., Cheng K.H., Huang J. (2006). Ceramide drives cholesterol out of the ordered lipid bilayer phase into the crystal phase in 1-palmitoyl-2-oleoyl-sn-glycero-3-phosphocholine/cholesterol/ceramide ternary mixtures. Biochemistry.

[9] Castro B.M., Silva L.C., Fedorov A., de Almeida R.F., Prieto M. (2009). Cholesterol-rich fluid membranes solubilize ceramide domains: Implications for the structure and dynamics of mammalian intracellular and plasma membranes. J. Biol. Chem..

[10] Hye A., Riddoch-Contreras J., Baird A.L., Ashton N.J., Bazenet C., Leung R., Westman E., Simmons A., Dobson R., Sattlecker M. (2014). Plasma proteins predict conversion to dementia from prodromal disease. Alzheimers Dement..

[11] Reisberg B., Ferris S.H., de Leon M.J., Crook T. (1982). The global deterioration scale for assessment of primary degenerative dementia. Am. J. Psychiatry.

[12] Mar J., Soto-Gordoa M., Arrospide A., Moreno-Izco F., Martínez-Lage P. (2015). Fitting the epidemiology and neuropathology of the early stages of Alzheimer’s disease to prevent dementia. Alzheimers Res.Ther..

[13] Quehenberger O., Armando A.M., Brown A.H., Milne S.B., Myers D.S., Merrill A.H., Bandyopadhyay S., Jones K.N., Kelly S., Shaner R.L. (2010). Lipidomics reveals a remarkable diversity of lipids in human plasma. J. Lipid Res..

[14] Lim W.F.L., Martins I.J., Martins R.N. (2014). Lipid metabolism and lipidomics: An emerging frontier in biology. J. Genet. Genom..

[15] Fonteh A.N., Fisher R.D. (2009). Combining lipidomics and proteomics of human cerebrospinal fluids. Methods Mol. Biol..

[16] Colsch B., Seyer A., Boudah S., Junot C. (2015). Lipidomic analysis of cerebrospinal fluid by mass spectrometry-based methods. J. Inherit. Metab. Dis..

[17] Martins I.J., Creegan R., Lim W.L.F., Martins R.N. (2013). Molecular insights into appetite control and neuroendocrine disease as risk factors for chronic diseases in Western countries. OJEMD.

[18] Martins I.J. (2013). Increased Risk for Obesity and Diabetes with Neurodegeneration in Developing Countries. J. Mol. Genet. Med..

[19] Fiandaca M.S., Zhong X., Cheema A.-K., Orquiza M.-H., Chidambaram S., Tan M.T., Gresenz C., FitzGerald K.T., Nalls M.A., Singleton A.B. (2015). Plasma 24-metabolite Panel Predicts Preclinical Transition to Clinical Stages of Alzheimer’s Disease. Front. Neurol..

[20] Mapstone M., Cheema A.K., Fiandaca M.S., Zhong X., Mhyre T.R., MacArthur L.H., Hall W.J., Fisher S.G., Peterson D.R., Haley J.M. (2014). Plasma phospholipids identify antecedent memory impairment in older adults. Nat. Med..

[21] Martins I.J. (2015). Overnutrition Determines LPS Regulation of Mycotoxin Induced Neurotoxicity in Neurodegenerative Diseases. Int. J. Mol. Sci..

[22] Deisenhammer F., Bartos A., Egg R., Gilhus N.E., Giovannoni G., Rauer S., Sellebjerg F., Tumani H., Gilhus N.E., Barnes M.P., Brainin M. (2011). Routine cerebrospinal fluid (CSF) analysis. European Handbook of Neurological Management.

[23] Martins I.J., Gupta V., Wilson A.C., Fuller S.J., Martins R.N. (2014). Interactions between Apo E and Amyloid Beta and their Relationship to Nutriproteomics and Neurodegeneration. Curr. Proteom..

[24] Liu C.C., Kanekiyo T., Xu H., Bu G. (2013). Apolipoprotein E and Alzheimer disease: Risk, mechanisms and therapy. Nat. Rev. Neurol..

[25] Stachowska E., Maciejewska D., Ossowski P., Drozd A., Ryterska K., Banaszczak M., Milkiewicz M., Raszeja-Wyszomirska J., Slebioda M., Milkiewicz P. (2013). Apolipoprotein E4 allele is associated with substantial changes in the plasma lipids and hyaluronic acid content in patients with nonalcoholic fatty liver disease. J. Physiol. Pharmacol..

[26] De Feo E., Cefalo C., Arzani D., Amore R., Landolfi R., Grieco A., Ricciardi W., Miele L., Boccia S. (2012). A case-control study on the effects of the apolipoprotein E genotypes in nonalcoholic fatty liver disease. Mol. Biol. Rep..

[27] Yin Y.-W., Sun Q.-Q., Zhang B.-B., Hu A.-M., Liu H.-L., Wang Q., Hou Z.Z. (2013). Association between Apolipoprotein E Gene Polymorphism and the Risk of Coronary Artery Disease in Chinese Population: Evidence from a Meta-Analysis of 40 Studies. PLoS ONE.

[28] Watson G.S., Craft S. (2003). The role of insulin resistance in the pathogenesis of Alzheimer’s disease: Implications for treatment. CNS Drugs.

[29] Schug T.T., Li X. (2011). Sirtuin 1 in lipid metabolism and obesity. Ann. Med..

[30] Purushotham A., Schug T.T., Xu Q., Surapureddi S., Guo X., Li X. (2009). Hepatocyte-specific deletion of SIRT1 alters fatty acid metabolism and results in hepatic steatosis and inflammation. Cell Metab..

[31] Maizel J., Xavier S., Chen J., Lin C.H., Vasko R., Goligorsky M.S. (2014). Sirtuin 1 ablation in endothelial cells is associated with impaired angiogenesis and diastolic dysfunction. Am. J. Physiol. Heart Circ. Physiol..

[32] Botti C., Caiafa I., Coppola A., Cuomo F., Miceli M., Altucci L., Cobellis G. (2014). SIRT1 inhibition affects angiogenic properties of human MSCs. Biomed. Res. Int..

[33] Potente M., Ghaeni L., Baldessari D., Mostoslavsky R., Rossig L., Dequiedt F., Haendeler J., Mione M., Dejana E., Alt F.W. (2007). SIRT1 controls endothelial angiogenic functions during vascular growth. Genes Dev..

[34] Hall J.A., Dominy J.E., Lee Y., Puigserver P. (2013). The sirtuin family’s role in aging and age-associated pathologies. J. Clin. Invest..

[35] Bonda D.J., Lee H.G., Camins A., Pallàs M., Casadesus G., Smith M.A., Zhu X. (2011). The sirtuin pathway in ageing and Alzheimer disease: Mechanistic and therapeutic considerations. Lancet Neurol..

[36] Martins I.J., Atta-ur-Rahman (2015). Nutritional and genotoxic stress contributes to diabetes and neurodegenerative diseases such as Parkinson’s and Alzheimer’s diseases. Frontiers in Clinical Drug Research—CNS and Neurological Disorders.

[37] Martins I.J. (2016). Anti-Aging Genes Improve Appetite Regulation and Reverse Cell Senescence and Apoptosis in Global Populations. Adv. Aging Res..

[38] Martins I.J. (2015). Unhealthy diets determine benign or toxic amyloid beta states and promote brain amyloid beta aggregation. Austin J. Clin. Neurol..

[39] Martins I.J. (2015). Diabetes and cholesterol dyshomeostasis involve abnormal α-synuclein and amyloid beta transport in neurodegenerative diseases. Austin Alzheimer’s J. Parkinson’s Dis..

[40] Ions L.J., Wakeling L.A., Bosomworth H.J., Hardyman J.E., Escolme S.M., Swan D.C., Valentine R.A., Mathers J.C., Ford D. (2013). Effects of Sirt1 on DNA methylation and expression of genes affected by dietary restriction. Age (Dordr.).

[41] Qiao L., Shao J. (2006). SIRT1 regulates adiponectin gene expression through Foxo1-C/enhancer-binding protein alpha transcriptional complex. J. Biol. Chem..

[42] Chihara Y., Rakugi H., Ishikawa K., Ikushima M., Maekawa Y., Ohta J., Kida I., Ogihara T. (2006). Klotho protein promotes adipocyte differentiation. Endocrinology.

[43] Jin Q., Zhang F., Yan T., Liu Z., Chunxi W., Ge X., Zhai Q. (2010). C/EBPα regulates SIRT1 expression during adipogenesis. Cell Res..

[44] Oka S., Alcendor R., Zhai P., Park J.Y., Shao D., Cho J., Yamamoto T., Tian B., Sadoshima J. (2011). PPARα-Sirt1 complex mediates cardiac hypertrophy and failure through suppression of the ERR transcriptional pathway. Cell Metab..

[45] Westmacott A., Burke Z.D., Oliver G., Slack J.M., Tosh D. (2006). C/EBPalpha and C/EBPbeta are markers of early liver development. Int. J Dev. Biol..

[46] Chau M.D., Gao J., Yang Q., Wu Z., Gromada J. (2010). Fibroblast growth factor 21 regulates energy metabolism by activating the AMPK-SIRT1-PGC-1alpha pathway. PNAS.

[47] Suzuki M., Uehara Y., Motomura-Matsuzaka K., Oki J., Koyama Y., Kimura M., Asada M., Komi-Kuramochi A., Oka S., Imamura T. (2008). betaKlotho is required for fibroblast growth factor (FGF) 21 signaling through FGF receptor (FGFR) 1c and FGFR3c. Mol. Endocrinol..

[48] Yie J., Wang W., Deng L., Tam L.T., Stevens J., Chen M.M., Li Y., Xu J., Lindberg R., Hecht R. (2012). Understanding the physical interactions in the FGF21/FGFR/β-Klotho complex: Structural requirements and implications in FGF21 signaling. Chem. Biol. Drug Des..

[49] Wolf I., Levanon-Cohen S., Bose S., Ligumsky H., Sredni B., Kanety H., Kuro-o M., Karlan B., Kaufman B., Koeffler H.P. (2008). Klotho: A tumor suppressor and a modulator of the IGF-1 and FGF pathways in human breast cancer. Oncogene.

[50] Piya M.K., Harte A.L., Chittari M.V., Tripathi G., Kumar S., McTernan P.G. (2013). FGF21 action on human adipose tissue compromised by reduced βKlotho and FGFR1 expression in type 2 diabetes mellitus. Endocr. Abstr..

[51] Bass J. (2013). Forever (FGF) 21. Nat. Med..

[52] Mäkelä J., Tselykh T.V., Maiorana F., Eriksson O., Do H.T., Mudò G., Korhonen L.T., Belluardo N., Lindholm D. (2014). Fibroblast growth factor-21 enhances mitochondrial functions and increases the activity of PGC-1α in human dopaminergic neurons via Sirtuin-1. Springerplus.

[53] Lundåsen T., Hunt M.C., Nilsson L.M., Sanyal S., Angelin B., Alexson S.E., Rudling M. (2007). PPARalpha is a key regulator of hepatic FGF21. Biochem. Biophys. Res. Commun..

[54] Akbar H., Batistel F., Drackley J.K., Loor J.J. (2015). Alterations in Hepatic *FGF21*, Co-Regulated Genes, and Upstream Metabolic Genes in Response to Nutrition, Ketosis and Inflammation in Peripartal Holstein Cows. PLoS ONE.

[55] Li Y., Wong K., Giles A., Jiang J., Lee J.W., Adams A.C., Kharitonenkov A., Yang Q., Gao B., Guarente L. (2014). Hepatic SIRT1 attenuates hepatic steatosis and controls energy balance in mice by inducing fibroblast growth factor 21. Gastroenterology.

[56] Xu J., Lloyd D.J., Hale C., Stanislaus S., Chen M., Sivits G., Vonderfecht S., Hecht R., Li Y.S., Lindberg R.A. (2009). Fibroblast growth factor 21 reverses hepatic steatosis, increases energy expenditure, and improves insulin sensitivity in diet-induced obese mice. Diabetes.

[57] Lin Z., Tian H., Lam K.S., Lin S., Hoo R.C., Konishi M., Itoh N., Wang Y., Bornstein S.R., Xu A., Li X. (2013). Adiponectin mediates the metabolic effects of FGF21 on glucose homeostasis and insulin sensitivity in mice. Cell Metab..

[58] Beenken A., Mohammadi M. (2009). The FGF family: Biology, pathophysiology and therapy. Nat. Rev. Drug Discov..

[59] Itoh N. (2014). FGF21 as a Hepatokine, Adipokine, and Myokine in Metabolism and Diseases. Front. Endocrinol..

[60] Liang Q., Zhong L., Zhang J., Wang Y., Bornstein S.R., Triggle C.R., Ding H., Lam K.S., Xu A. (2014). FGF21 maintains glucose homeostasis by mediating the cross talk between liver and brain during prolonged fasting. Diabetes.

[61] Inagaki T., Lin V.Y., Goetz R., Mohammadi M., Mangelsdorf D.J., Kliewer S.A. (2008). Inhibition of Growth Hormone Signaling by the Fasting-Induced Hormone FGF21. Cell Metab..

[62] Holland W.L., Adams A.C., Brozinick J.T., Bui H.H., Miyauchi Y., Kusminski C.M., Bauer S.M., Wade M., Singhal E., Cheng C.C. (2013). An FGF21-adiponectin-ceramide axis controls energy expenditure and insulin action in mice. Cell Metab..

[63] Tan B.K., Hallschmid M., Adya R., Kern W., Lehnert H., Randeva H.S. (2011). Fibroblast growth factor 21 (FGF21) in human cerebrospinal fluid: relationship with plasma FGF21 and body adiposity. Diabetes.

[64] Tao C., Sifuentes A., Holland W.L. (2014). Regulation of glucose and lipid homeostasis by adiponectin: Effects on hepatocytes, pancreatic β cells and adipocytes. Best Pract. Res. Clin. Endocrinol. Metab..

[65] Holland W.L., Miller R.A., Wang Z.V., Sun K., Barth B.M., Bui H.H., Davis K.E., Bikman B.T., Halberg N., Rutkowski J.M. (2011). Receptor-mediated activation of ceramidase activity initiates the pleiotropic actions of adiponectin. Nat. Med..

[66] Okua H., Matsuuraa F., Kosekia M., Sandovala J.C., Yuasa-Kawasea M., Tsubakio-Yamamotoa K., Masudab D., Maedab N., Ohamaa T., Ishigamic M. (2007). Adiponectin deficiency suppresses ABCA1 expression and ApoA-I synthesis in the liver. FEBS Lett..

[67] Dantas K.C., Bydlowski S.P., Novak E.M. (2006). Study of activity transcription factors C/EBPα in region-53 to -33 of promoter apolipoprotein B gene. Rev. Bras. Ciências Farm. Braz. J. Pharm. Sci..

[68] Bhalla S., Ozalp C., Fang S., Xiang L., Kemper J.K. (2004). Ligand-activated pregnane X receptor interferes with HNF-4 signaling by targeting a common coactivator PGC-1alpha. Functional implications in hepatic cholesterol and glucose metabolism. J. Biol. Chem..

[69] Rhee J., Ge H., Yang W., Fan M., Handschin C., Cooper M., Lin J., Li C., Spiegelman B.M. (2006). Partnership of PGC-1alpha and HNF4alpha in the regulation of lipoprotein metabolism. J. Biol. Chem..

[70] Mogilenko D.A., Dizhe E.B., Shavva V.S., Lapikov I.A., Orlov S.V., Perevozchikov A.P. (2009). Role of the nuclear receptors HNF4 alpha, PPAR alpha, and LXRs in the TNF alpha-mediated inhibition of human apolipoprotein A-I gene expression in HepG2 cells. Biochemistry.

[71] Yamamoto T., Shimano H., Nakagawa Y., Ide T., Yahagi N., Matsuzaka T., Nakakuki M., Takahashi A., Suzuki H., Sone H. (2004). SREBP-1 interacts with hepatocyte nuclear factor-4 alpha and interferes with PGC-1 recruitment to suppress hepatic gluconeogenic genes. J. Biol. Chem..

[72] Asher G., Gatfield D., Stratmann M., Reinke H., Dibner C., Kreppel F., Mostoslavsky R., Alt F.W., Schibler U. (2008). SIRT1 regulates circadian clock gene expression through PER2 deacetylation. Cell.

[73] Jung-Hynes B., Ahmad N. (2009). SIRT1 controls circadian clock circuitry and promotes cell survival: A connection with age-related neoplasms. FASEB J..

[74] Loh D.H., Dragich J.M., Kudo T., Schroeder A.M., Nakamura T.J., Waschek J.A., Block G.D., Colwell C.S. (2011). Effects of vasoactive intestinal peptide genotype on circadian gene expression in the suprachiasmatic nucleus and peripheral organs. J. Biol. Rhythms.

[75] Piggins H.D., Cutler D.J. (2003). The roles of vasoactive intestinal polypeptide in the mammalian circadian clock. J. Endocrinol..

[76] Aton S.J., Colwell C.S., Harmar A.J., Waschek J., Herzog E.D. (2005). Vasoactive intestinal polypeptide mediates circadian rhythmicity and synchrony in mammalian clock neurons. Nat. Neurosci..

[77] Nussdorfer G.G., Malendowicz L.K. (1998). Role of VIP, PACAP, and related peptides in the regulation of the hypothalamo-pituitary-adrenal axis. Peptides.

[78] Gozes I., Zamostiano R., Pinhasov A., Bassan M., Giladi E., Steingart R.A., Brenneman D.E. (2000). A novel VIP responsive gene. Activity dependent neuroprotective protein. Ann. N. Y. Acad. Sci..

[79] Gozes I., Divinsky I., Pilzer I., Fridkin M., Brenneman D.E., Spier A.D. (2003). From vasoactive intestinal peptide (VIP) through activity-dependent neuroprotective protein (ADNP) to NAP: A view of neuroprotection and cell division. J. Mol. Neurosci..

[80] Gozes I. (2007). Activity-dependent neuroprotective protein: From gene to drug candidate. Pharmacol. Ther..

[81] Yang M.H., Yang Y.H., Lu C.Y., Jong S.B., Chen L.J., Lin Y.F., Wu S.J., Chu P.Y., Chung T.W., Tyan Y.C. (2012). Activity-dependent neuroprotector homeobox protein: A candidate protein identified in serum as diagnostic biomarker for Alzheimer’s disease. J. Proteom..

[82] Gozes I., Bardea A., Reshef A., Zamostiano R., Zhukovsky S., Rubinraut S., Fridkin M., Brenneman D.E. (1996). Neuroprotective strategy for Alzheimer disease: intranasal administration of a fatty neuropeptide. PNAS.

[83] White C.M., Ji S., Cai H., Maudsley S., Martin B. (2010). Therapeutic potential of vasoactive intestinal peptide and its receptors in neurological disorders. CNS Neurol. Disord. Drug Targets.

[84] Hawley C.M., Duggan K.A., Macdonald G.J., Shelley S. (1991). Oral sodium regulates extrahepatic metabolism of vasoactive intestinal peptide. Clin. Sci..

[85] Davis R.E., Shelley S., MacDonald G.J., Duggan K.A. (1992). The effects of a high sodium diet on the metabolism and secretion of vasoactive intestinal peptide in the rabbit. J. Physiol..

[86] Martins I.J. (2014). The Global Obesity Epidemic is Related to Stroke, Dementia and Alzheimer’s disease. JSM Alzheimer’s Dis. Relat. Dement..

[87] Fuenmayor N., Moreira E., Cubeddu L.X. (1998). Salt sensitivity is associated with insulin resistance in essential hypertension. Am. J. Hypertens..

[88] Rocchini A.P. (1994). The relationship of sodium sensitivity to insulin resistance. Am. J. Med. Sci..

[89] Rocchini A.P., Katch V., Kveselis D., Moorehead C., Martin M., Lampman R., Gregory M. (1989). Insulin and renal sodium retention in obese adolescents. Hypertension.

[90] Sawmiller D.R., Henning R.J., Cuevas J., Dehaven W.I., Vesely D.L. (2004). Coronary vascular effects of vasoactive intestinal peptide in the isolated perfused rat heart. Neuropeptides.

[91] Dogrukol-Ak D., Tore F., Tuncel N. (2004). Passage of VIP/PACAP/secretin family across the blood-brain barrier: Therapeutic effects. Curr. Pharm. Des..

[92] Dogrukol-Ak D., Banks W.A., Tuncel N., Tuncel M. (2009). Passage of vasoactive intestinal peptide across the blood-brain barrier. Peptides.

[93] Ji H., Zhang Y., Liu Y., Shen X.D., Gao F., Nguyen T.T., Busuttil R.W., Waschek J.A., Kupiec-Weglinski J.W. (2013). Vasoactive intestinal peptide attenuates liver ischemia/reperfusion injury in mice via the cyclic adenosine monophosphate-protein kinase a pathway. Liver Transplant..

[94] Xu Y., Zalzala M., Xu J., Li Y., Yin L., Zhang Y. (2015). A metabolic stress-inducible miR-34a-HNF4α pathway regulates lipid and lipoprotein metabolism. Nat. Commun..

[95] Morgado A.L., Xavier J.M., Dionísio P.A., Ribeiro M.F., Dias R.B., Sebastião A.M., Solá S., Rodrigues C.M. (2015). MicroRNA-34a Modulates Neural Stem Cell Differentiation by Regulating Expression of Synaptic and Autophagic Proteins. Mol. Neurobiol..

[96] Wang J., Cheng H., Li X., Lu W., Wang K., Wen T. (2013). Regulation of neural stem cell differentiation by transcription factors HNF4-1 and MAZ-1. Mol. Neurobiol..

[97] Jung D., Kullak-Ublick G.A. (2003). Hepatocyte nuclear factor 1 alpha: A key mediator of the effect of bile acids on gene expression. Hepatology.

[98] Sen N., Satija Y.K., Das S. (2011). PGC-1α, a Key Modulator of p53, Promotes Cell Survival upon Metabolic Stress. Mol. Cell.

[99] Kamiya A., Inoue Y., Gonzalez F.J. (2003). Role of the hepatocyte nuclear factor 4alpha in control of the pregnane X receptor during fetal liver development. Hepatology.

[100] (2016). ADNP activity-dependent neuroprotector homeobox [Homo sapiens (human)] Gene ID: 23394. http://www.ncbi.nlm.nih.gov/gene/23394.

[101] Lu P., Liu J., Melikishvili M., Fried M.G., Chi Y.I. (2008). Crystallization of hepatocyte nuclear factor 4 alpha (HNF4 alpha) in complex with the HNF1 alpha promoter element. Acta Crystallogr. Sect. F Struct. Biol. Cryst. Commun..

[102] Grimm A.A., Brace C.S., Wang T., Stormo G.D., Imai S. (2011). A nutrient-sensitive interaction between Sirt1 and HNF-1α regulates Crp expression. Aging Cell.

[103] Ellard S. (2000). Hepatocyte nuclear factor 1 alpha (HNF-1 alpha) mutations in maturity-onset diabetes of the young. Hum. Mutat..

[104] Maetzler W., Tian Y., Baur S.M., Gauger T., Odoj B., Schmid B. (2012). Serum and Cerebrospinal Fluid Levels of Transthyretin in Lewy Body Disorders with and without Dementia. PLoS ONE.

[105] Woodward D.K., Hatton J., Ensom M.H., Young B., Dempsey R., Clifton G.D. (1998). Alpha1-acid glycoprotein concentrations and cerebrospinal fluid drug distribution after subarachnoid hemorrhage. Pharmacotherapy.

[106] Mattsson N., Insel P., Nosheny R., Trojanowski J.Q., Shaw L.M., Jack C.R., Weiner M. (2014). Effects of cerebrospinal fluid proteins on brain atrophy rates in cognitively healthy older adults. Neurobiol. Aging.

[107] Kaynar M.Y., Tanriverdi T., Kafadar A.M., Kacira T., Uzun H., Aydin S., Gumustas K., Dirican A., Kuday C. (2004). Detection of soluble intercellular adhesion molecule-1 and vascular cell adhesion molecule-1 in both cerebrospinal fluid and serum of patients after aneurysmal subarachnoid hemorrhage. J. Neurosurg..

[108] Jongen P.J., Doesburg W.H., Ibrahim-Stappers J.L., Lemmens W.A., Hommes O.R., Lamers K.J. (2000). Cerebrospinal fluid C3 and C4 indexes in immunological disorders of the central nervous system. Acta Neurol. Scand..

[109] Kuncl R.W., Bilak M.M., Bilak S.R., Corse A.M., Royal W., Becerra S.P. (2002). Pigment epithelium-derived factor is elevated in CSF of patients with amyotrophic lateral sclerosis. J. Neurochem..

[110] Pearl G.S., Mullins R.E. (1985). Alpha 1-antitrypsin in cerebrospinal fluid of patients with neurologic diseases. Arch. Neurol..

[111] Rentzos M., Nikolaou C., Rombos A., Boufidou F., Zoga M., Dimitrakopoulos A., Tsoutsou A., Vassilopoulos D. (2007). RANTES levels are elevated in serum and cerebrospinal fluid in patients with amyotrophic lateral sclerosis. Amyotroph. Lateral Scler..

[112] Semba R.D., Moghekar A.R., Hu J., Sun K., Turner R., Ferrucci L., O’Brien R. (2014). Klotho in the cerebrospinal fluid of adults with and without Alzheimer's disease. Neurosci. Lett..

[113] Shah D.J., Rohlfing F., Anand S., Johnson W.E., Alvarez M.T., Cobell J., King J., Young S.A., Kauwe J.S., Graves S.W. (2015). Discovery and Subsequent Confirmation of Novel Serum Biomarkers Diagnosing Alzheimer’s Disease. Alzheimers Dis..

[114] Bilic E., Bilic E., Rudan I., Kusec V., Zurak N., Delimar D., Zagar M. (2006). Comparison of the growth hormone, IGF-1 and insulin in cerebrospinal fluid and serum between patients with motor neuron disease and healthy controls. Eur. J Neurol..

[115] Kos K., Harte A.L., da Silva N.F., Tonchev A., Chaldakov G., James S., Snead D.R., Hoggart B., O’Hare J.P., McTernan P.G. (2007). Adiponectin and resistin in human cerebrospinal fluid and expression of adiponectin receptors in the human hypothalamus. J. Clin. Endocrinol. Metab..

[116] Pirttilä T., Vanhatalo S., Turpeinen U., Riikonen R. (2004). Cerebrospinal fluid insulin-like growth factor-1, insulin growth factor binding protein-2 or nitric oxide are not increased in MS or ALS. Acta Neurol. Scand..

[117] Ji L., Zhao X., Hua Z. (2015). Potential therapeutic implications of gelsolin in Alzheimer’s disease. J. Alzheimers Dis..

[118] Güntert A., Campbell J., Saleem M., O’Brien D.P., Thompson A.J., Byers H.L., Ward M.A., Lovestone S. (2010). Plasma gelsolin is decreased and correlates with rate of decline in Alzheimer's disease. J. Alzheimers Dis..

[119] Restituto P., Colina I., Varo J.J., Varo N. (2010). Adiponectin diminishes platelet aggregation and sCD40L release. Potential role in the metabolic syndrome. Am. J. Physiol. Endocrinol. Metab..

[120] Wang W.Q., Zhang H.F., Gao G.X., Bai Q.X., Li R., Wang X.M. (2011). Adiponectin inhibits hyperlipidemia-induced platelet aggregation via attenuating oxidative/nitrative stress. Physiol. Res..

[121] Carnevale R., Pastori D., Peruzzi M., de Falco E., Chimenti I., Biondi-Zoccai G., Greco E., Marullo A.G.M., Nocella C., Violi F. (2014). Total Adiponectin Is Inversely Associated with Platelet Activation and CHA2DS2-VASc Score in Anticoagulated Patients with Atrial Fibrillation. Mediat. Inflamm..

[122] Ouchi N., Kobayashi H., Kihara S., Kumada M., Sato K., Inoue T., Funahashi T., Walsh K. (2004). Adiponectin stimulates angiogenesis by promoting cross-talk between AMP-activated protein kinase and Akt signaling in endothelial cells. J. Biol. Chem..

[123] Wang Y., Xu L.Y., Lam K.S., Lu G., Cooper G.J., Xu A. (2006). Proteomic characterization of human serum proteins associated with the fat-derived hormone adiponectin. Proteomics.

[124] Son S.M., Nam D.W., Cha M.Y., Kim K.H., Byun J., Ryu H., Mook-Jung I. (2015). Thrombospondin-1 prevents amyloid beta-mediated synaptic pathology in Alzheimer’s disease. Neurobiol. Aging.

[125] Rama Rao K.V., Curtis K.M., Johnstone J.T., Norenberg M.D. (2013). Amyloid-β inhibits thrombospondin 1 release from cultured astrocytes: effects on synaptic protein expression. J. Neuropathol. Exp. Neurol..

[126] Liauw J., Hoang S., Choi M., Eroglu C., Choi M., Sun G.H., Percy M., Wildman-Tobriner B., Bliss T., Guzman R.G. (2008). Thrombospondins 1 and 2 are necessary for synaptic plasticity and functional recovery after stroke. J. Cereb. Blood Flow Metab..

[127] Kounnas M.Z., Morris R.E., Thompson M.R., FitzGerald D.J., Strickland D.K., Saelinger C.B. (1992). The alpha 2-macroglobulin receptor/low density lipoprotein receptor-related protein binds and internalizes Pseudomonas exotoxin A. J. Biol. Chem..

[128] Naganuma H., Satoh E., Asahara T., Amagasaki K., Watanabe A., Satoh H., Kuroda K., Zhang L., Nukui H. (2004). Quantification of thrombospondin-1 secretion and expression of alphavbeta3 and alpha3beta1 integrins and syndecan-1 as cell-surface receptors for thrombospondin-1 in malignant glioma cells. J. Neurooncol..

[129] Resovi A., Pinessi D., Chiorino G., Taraboletti G. (2014). Current understanding of the thrombospondin-1 interactome. Matrix Biol..

[130] Choi K.Y., Kim D.B., Kim M.J., Kwon B.J., Chang S.Y., Jang S.W., Cho E.J., Rho T.H., Kim J.H. (2012). Higher plasma thrombospondin-1 levels in patients with coronary artery disease and diabetes mellitus. Korean Circ. J..

[131] Roberts D.D., Miller T.W., Rogers N.M., Yao M., Isenberg J.S. (2012). The matricellular protein thrombospondin-1 globally regulates cardiovascular function and responses to stress via CD47. Matrix Biol..

[132] Kong P., Cavalera M., Frangogiannis N.G. (2014). The role of thrombospondin (TSP)-1 in obesity and diabetes. Adipocyte.

[133] Maier K.G., Han X., Sadowitz B., Gentile K.L., Middleton F.A., Gahtan V. (2010). Thrombospondin-1: A proatherosclerotic protein augmented by hyperglycemia. J. Vasc. Surg..

[134] Isenberg J.S., Romeo M.J., Yu C., Yu C.K., Nghiem K., Monsale J., Rick M.E., Wink D.A., Frazier W.A., Roberts D.D. (2008). Thrombospondin-1 stimulates platelet aggregation by blocking the antithrombotic activity of nitric oxide/cGMP signaling. Blood.

[135] Starlinger P., Moll H.P., Assinger A., Nemeth C., Hoetzenecker K., Gruenberger B., Gruenberger T., Kuehrer I., Schoppmann S.F., Gnant M. (2010). Thrombospondin-1: A unique marker to identify *in vitro* platelet activation when monitoring *in vivo* processes. J. Thromb. Haemost..

[136] Lawler P.R., Lawler J. (2012). Molecular basis for the regulation of angiogenesis by thrombospondin-1 and -2. Cold Spring Harb. Perspect. Med..

[137] Maimaitiyiming H., Clemons K., Zhou Q., Norman H., Wang S. (2015). Thrombospondin1 Deficiency Attenuates Obesity-Associated Microvascular Complications in ApoE−/− Mice. PLoS ONE.

[138] Dabrowska K., Lewandowski E., Skowronska-Jozwiak A., Brona A., Milewicz A., Lewinski A. (2012). Serum concentrations of thrombospondin-1 and adiponectin in patients with hyperthyroidism before and after normalisation of thyroid function. Endocr. Abstr..

[139] Daniel C., Schaub K., Amann K., Lawler J., Hugo C. (2007). Thrombospondin-1 is an endogenous activator of TGF-beta in experimental diabetic nephropathy *in vivo*. Diabetes.

[140] Murphy-Ullrich J.E., Poczatek M. (2000). Activation of latent TGF-beta by thrombospondin-1: Mechanisms and physiology. Cytokine Growth Factor Rev..

[141] Ribeiro S.M., Poczatek M., Schultz-Cherry S., Villain M., Murphy-Ullrich J.E. (1999). The activation sequence of thrombospondin-1 interacts with the latency-associated peptide to regulate activation of latent transforming growth factor-beta. J. Biol. Chem..

[142] Daniel C., Wiede J., Krutzsch H.C., Ribeiro S.M., Roberts D.D., Murphy-Ullrich J.E., Hugo C. (2004). Thrombospondin-1 is a major activator of TGF-beta in fibrotic renal disease in the rat *in vivo*. Kidney Int..

[143] Hayashi H., Sakai K., Baba H., Sakai T. (2012). Thrombospondin-1 is a novel negative regulator of liver regeneration after partial hepatectomy via TGF-β1 activation in mice. Hepatology.

[144] Tesseur I., Zou K., Esposito L., Bard F., Berber E., Can J.V., Lin A.H., Crews L., Tremblay P., Mathews P. (2006). Deficiency in neuronal TGF-beta signaling promotes neurodegeneration and Alzheimer’s pathology. J. Clin. Invest..

[145] Wyss-Coray T., Lin C., Yan F., Yu G.Q., Rohde M., McConlogue L., Masliah E., Mucke L. (2001). TGF-beta1 promotes microglial amyloid-beta clearance and reduces plaque burden in transgenic mice. Nat. Med..

[146] Das P., Golde T. (2006). Dysfunction of TGF-beta signaling in Alzheimer’s disease. J. Clin. Invest..

[147] Wyss-Coray T., Masliah E., Mallory M., McConlogue L., Johnson-Wood K., Lin C., Mucke L. (1997). Amyloidogenic role of cytokine TGF-beta1 in transgenic mice and in Alzheimer’s disease. Nature.

[148] Yang L., Roh Y.S., Song J., Zhang B., Liu C., Loomba R., Seki E. (2014). Transforming growth factor beta signaling in hepatocytes participates in steatohepatitis through regulation of cell death and lipid metabolism in mice. Hepatology.

[149] Dooley S., Ten Dijke P. (2012). TGF-β in progression of liver disease. Cell Tissue Res..

[150] Wei Y., Tian Q., Zhao X., Wang X. (2015). Serum transforming growth factor beta 3 predicts future development of nonalcoholic fatty liver disease. Int. J. Clin. Exp. Med..

[151] Chao C.C., Hu S., Frey W.H., Ala T.A., Tourtellotte W.W., Peterson P.K. (1994). Transforming growth factor beta in Alzheimer’s disease. Clin. Diagn. Lab. Immunol..

[152] Sinha M., Jang Y.C., Oh J., Khong D., Wu E.Y., Manohar R., Miller C., Regalado S.G., Loffredo F.S., Pancoast J.R. (2014). Restoring systemic GDF11 levels reverses age-related dysfunction in mouse skeletal muscle. Science.

[153] Ge G., Hopkins D.R., Ho W.-B., Greenspan D.S. (2005). GDF11 Forms a Bone Morphogenetic Protein 1-Activated Latent Complex That Can Modulate Nerve Growth Factor-Induced Differentiation of PC12 Cells. Mol. Cell. Biol..

[154] Ganesh V., Hettiarachchy N.S. (2012). Nutriproteomics: A promising tool to link diet and diseases in nutritional research. Biochim. Biophys. Acta.

[155] Sénéchal S., Kussmann M. (2011). Nutriproteomics: Technologies and applications for identification and quantification of biomarkers and ingredients. Proc. Nutr. Soc..

[156] Sauer S., Luge T. (2015). Nutriproteomics: Facts, concepts, and perspectives. Proteomics.

[157] Hu W.T., Chen-Plotkin A., Arnold S.E., Grossman M., Clark C.M., Shaw L.M., Pickering E., Kuhn M., Chen Y., McCluskey L. (2010). Novel CSF biomarkers for Alzheimer’s disease and mild cognitive impairment. Acta Neuropathol..

[158] Neumeier M., Weigert J., Buettner R., Wanninger J., Schäffler A., Müller A.M., Killian S., Sauerbruch S., Schlachetzki F., Steinbrecher A. (2007). Detection of adiponectin in cerebrospinal fluid in humans. Am. J. Physiol. Endocrinol. Metab..

[159] Une K., Takei Y.A., Tomita N., Asamura T., Ohrui T., Furukawa K., Arai H. (2011). Adiponectin in plasma and cerebrospinal fluid in MCI and Alzheimer’s disease. Eur. J. Neurol..

[160] Kusminski C.M., McTernan P.G., Schraw T., Kos K., O’Hare J.P., Ahima R., Kumar S., Scherer P.E. (2007). Adiponectin complexes in human cerebrospinal fluid: Distinct complex distribution from serum. Diabetologia.

[161] Choe Y.G., Jin W., Cho Y.K., Chung W.G., Kim H.J., Jeon W.K., Kim B.I. (2013). Apolipoprotein B/AI ratio is independently associated with non-alcoholic fatty liver disease in nondiabetic subjects. J. Gastroenterol. Hepatol..

[162] Martins I.J. (2015). LPS Regulates Apolipoprotein E and Aβ Interactions with Effects on Acute Phase Proteins and Amyloidosis. Adv. Aging Res..

[163] De Almeida R.L., Constancio J., Vendramini R.C., Fracasso J.F., Menani J.V. (2011). Lipopolysaccharide reduces sodium intake and sodium excretion in dehydrated rats. Physiol. Behav..

[164] Martins I.J. (2015). Diabetes and organ dysfunction in the developing and developed. World Glob. J. Med. Res. F Dis..

[165] Affarah H.B., Hall W.D., Heymsfield S.B., Kutner M., Wells J.O. (1986). High-carbohydrate diet: antinatriuretic and blood pressure response in normal men. Am. J. Clin. Nutr..

[166] Matsuura F., Oku H., Koseki M., Sandoval J.C., Yuasa-Kawase M., Tsubakio-Yamamoto K., Masuda D., Maeda N., Tsujii K., Ishigami M. (2007). Adiponectin accelerates reverse cholesterol transport by increasing high density lipoprotein assembly in the liver. Biochem. Biophys. Res. Commun..

[167] Liang B., Wang X., Guo X., Yang Z., Bai R., Liu M., Xiao C., Bian Y. (2015). Adiponectin upregulates ABCA1 expression through liver X receptor alpha signaling pathway in RAW 264.7 macrophages. Int. J. Clin. Exp. Pathol..

[168] Inoue M., Jiang Y., Tokunaga M., Martinez-Santibañez G., Geletka L., Lumeng C.N., Buchner D.A., Chun T.H. (2013). Thrombospondin 1 mediates high-fat diet-induced muscle fibrosis and insulin resistance in male mice. Endocrinology.

[169] Cui W., Maimaitiyiming H., Qi X., Norman H., Wang S. (2013). Thrombospondin 1 mediates renal dysfunction in a mouse model of high-fat diet-induced obesity. Am. J. Physiol. Renal Physiol..

[170] Martins I.J. (2015). Nutritional Diets Accelerate Amyloid Beta Metabolism and Prevent the Induction of Chronic Diseases and Alzheimer’s Disease.

[171] Naito T., Masaki T., Nikolic-Paterson D.J., Tanji C., Yorioka N., Kohno N. (2004). Angiotensin II induces thrombospondin-1 production in human mesangial cells via p38 MAPK and JNK: A mechanism for activation of latent TGF-beta1. Am. J. Physiol. Renal Physiol..

[172] Chua C.C., Hamdy R.C., Chua B.H. (1997). Regulation of thrombospondin-1 production by angiotensin II in rat heart endothelial cells. Biochim. Biophys. Acta.

[173] Lutz J., Huwiler K.G., Fedczyna T., Lechman T.S., Crawford S., Kinsella T.R., Pachman L.M. (2002). Increased plasma thrombospondin-1 (TSP-1) levels are associated with the TNF alpha-308A allele in children with juvenile dermatomyositis. Clin. Immunol..

[174] Rege T.A., Stewart J., Dranka B., Benveniste E.N., Silverstein R.L., Gladson C.L. (2009). Thrombospondin-1-induced apoptosis of brain microvascular endothelial cells can be mediated by TNF-R1. J. Cell. Physiol..

[175] McMorrow J.P., Crean D., Gogarty M., Smyth A., Connolly M., Cummins E., Veale D., Fearon U., Tak P.P., Fitzgerald O. (2013). Tumor necrosis factor inhibition modulates thrombospondin-1 expression in human inflammatory joint disease through altered NR4A2 activity. Am. J. Pathol..

[176] Belarbi K., Jopson T., Tweedie D., Arellano C., Luo W., Greig N.H., Rosi S. (2012). TNF-α protein synthesis inhibitor restores neuronal function and reverses cognitive deficits induced by chronic neuroinflammation. J. Neuroinflamm..

[177] Tobinick E., Gross H., Weinberger A., Cohen H. (2006). TNF-α modulation for treatment of Alzheimer’s disease: A 6-month pilot study. MedGenMed.

[178] Woo Y.-C., Tso A.W.K., Xu A., Law L.S.C., Fong C.H.Y., Lam T.-H. (2012). Combined Use of Serum Adiponectin and Tumor Necrosis Factor-Alpha Receptor 2 Levels Was Comparable to 2-Hour Post-Load Glucose in Diabetes Prediction. PLoS ONE.

[179] Masaki T., Chiba S., Tatsukawa H., Yasuda T., Noguchi H., Seike M., Yoshimatsu H. (2004). Adiponectin protects LPS-induced liver injury through modulation of TNF-α in KK-Ay obese mice. Hepatology.

[180] Zhang H., Wu L.-M., Wu J. (2011). Cross-Talk between Apolipoprotein E and Cytokines. Mediat. Inflamm..

[181] Laskowitz D.T., Goel S., Bennett E.R., Matthew W.D. (1997). Apolipoprotein E suppresses glial cell secretion of TNF alpha. J. Neuroimmunol..

[182] Song H., Saito K., Fujigaki S., Noma A., Ishiguro H., Nagatsu T., Seishima M. (1998). IL-1 beta and TNF-alpha suppress apolipoprotein (apo) E secretion and apo A-I expression in HepG2 cells. Cytokine.

[183] Díaz-Delfín J., Hondares E., Iglesias R., Giralt M., Caelles C., Villarroya F. (2012). TNF-α represses β-Klotho expression and impairs FGF21 action in adipose cells: involvement of JNK1 in the FGF21 pathway. Endocrinology.

[184] Feingold K.R., Grunfeld C., Heuer J.G., Gupta A., Cramer M., Zhang T., Shigenaga J.K., Patzek S.M., Chan Z.W., Moser A. (2012). FGF21 is increased by inflammatory stimuli and protects leptin-deficient ob/ob mice from the toxicity of sepsis. Endocrinology.

[185] Woo Y.C., Xu A., Wang Y., Lam K.S. (2013). Fibroblast growth factor 21 as an emerging metabolic regulator: Clinical perspectives. Clin. Endocrinol..

[186] Wang W.F., Li S.M., Ren G.P., Zheng W., Lu Y.J., Yu Y.H., Xu W.J., Li T.H., Zhou L.H., Liu Y. (2015). Recombinant murine fibroblast growth factor 21 ameliorates obesity-related inflammation in monosodium glutamate-induced obesity rats. Endocrine.

[187] Zhou Z., Kang X., Jiang Y., Song Z., Feng W., McClain C.J., Kang Y.J. (2007). Preservation of hepatocyte nuclear factor-4alpha is associated with zinc protection against TNF-alpha hepatotoxicity in mice. Exp. Biol. Med..

[188] Von Bülow V., Dubben S., Engelhardt G., Hebel S., Plümäkers B., Heine H., Rink L., Haase H. (2007). Zinc-dependent suppression of TNF-alpha production is mediated by protein kinase A-induced inhibition of Raf-1, I kappa B kinase beta, and NF-kappa B. J. Immunol..

[189] Meerarani P., Ramadass P., Toborek M., Bauer H.C., Bauer H., Hennig B. (2000). Zinc protects against apoptosis of endothelial cells induced by linoleic acid and tumor necrosis factor alpha. Am. J. Clin. Nutr..

[190] Fordham J.B., Hua J., Morwood S.R., Schewitz-Bowers L.P., Copland D.A., Dick A.D., Nicholson L.B. (2012). Environmental conditioning in the control of macrophage thrombospondin-1 production. Sci. Rep..

[191] Gokyu M., Kobayashi H., Nanbara H., Sudo T., Ikeda Y., Suda T., Izumi Y. (2014). Thrombospondin-1 production is enhanced by Porphyromonas gingivalis lipopolysaccharide in THP-1 cells. PLoS ONE.

[192] Lu W., Jiang J.P., Hu J., Wang J., Zheng M.Z. (2015). Curcumin protects against lipopolysaccharide-induced vasoconstriction dysfunction via inhibition of thrombospondin-1 and transforming growth factor-β1. Exp. Ther. Med..

[193] De Haas C.J., van Leeuwen H.J., Verhoef J., van Kessel K.P., van Strijp J.A. (2000). Analysis of lipopolysaccharide (LPS)-binding characteristics of serum components using gel filtration of FITC-labeled LPS. J. Immunol. Methods.

[194] Wollenberg G.K., LaMarre J., Rosendal S., Gonias S.L., Hayes M.A. (1991). Binding of tumor necrosis factor alpha to activated forms of human plasma alpha 2 macroglobulin. Am. J. Pathol..

[195] Webb D.J., Gonias S.L. (1998). A modified human alpha 2-macroglobulin derivative that binds tumor necrosis factor-alpha and interleukin-1 beta with high affinity *in vitro* and reverses lipopolysaccharide toxicity *in vivo* in mice. Lab. Invest..

[196] Gourine A.V., Gourine V.N., Tesfaigzi Y., Caluwaerts N., Van Leuven F., Kluger M.J. (2002). Role of alpha(2)-macroglobulin in fever and cytokine responses induced by lipopolysaccharide in mice. Am. J. Physiol. Regul. Integr. Comp. Physiol..

[197] Cho S.M., Kim H.V., Lee S., Kim H.Y., Kim W., Kim T.S., Kim D.J., Kim Y.S. (2014). Correlations of amyloid-β concentrations between CSF and plasma in acute Alzheimer mouse model. Sci. Rep..

[198] Johanson C.E., Duncan J.A., Klinge P.M., Brinker T., Stopa E.G., Silverberg G.D. (2008). Multiplicity of cerebrospinal fluid functions: New challenges in health and disease. Cerebrospinal Fluid Res..

